# Efficacy and safety of inhaled budesonide on prevention of acute mountain sickness during emergent ascent: a meta-analysis of randomized controlled trials

**DOI:** 10.1186/s12873-020-00329-8

**Published:** 2020-05-13

**Authors:** Gaurav Nepal, Jayant Kumar Yadav, Jessica Holly Rehrig, Niroj Bhandari, Santosh Baniya, Rakesh Ghimire, Narayan Mahotra

**Affiliations:** 1 Maharajgunj Medical Campus, Tribhuvan University Institute of Medicine, Kathmandu, Nepal; 2 Mountain Medicine Society of Nepal, Kathmandu, Nepal; 3 University of New England College of Osteopathic Medicine, Biddeford, ME USA; 4 Kathmandu University School of Medical Sciences, Panauti, Nepal

**Keywords:** Acute Mountain sickness, Emergency ascent, High altitude illness, Inhaled budesonide, Budesonide, Disaster, Rescue, Meta-analysis

## Abstract

**Background:**

Acute Mountain Sickness (AMS) is a pathophysiologic process that occurs in non-acclimated susceptible individuals rapidly ascending to high-altitude. Barometric pressure falls at high altitude and it translates to a decreased partial pressure of alveolar oxygen (PAO2) and arterial oxygen (PaO2). A gradual staged ascent with sufficient acclimatization can prevent AMS but emergent circumstances requiring exposure to rapid atmospheric pressure changes – such as for climbers, disaster or rescue team procedures, and military operations – establishes a need for effective prophylactic medications. This systematic review and meta-analysis aim to analyze the incidence of AMS during emergent ascent of non-acclimatized individuals receiving inhaled budesonide compared to placebo.

**Methods:**

This current meta-analysis was conducted according to the guidance of the Preferred Reporting Items for Systematic Reviews and Meta-Analyses statement. We searched PubMed, Google Scholar and Embase for relevant studies. The efficacy of budesonide in reducing incidence of AMS was evaluated by calculating the pooled ORs and 95% CIs. The efficacy of budesonide in maintaining hemoglobin-oxygen saturation was evaluated by calculating standard mean difference (SMD) and 95% confidence intervals.

**Results:**

We found that at high altitude, inhaled budesonide was effective in reducing the incidence of mild AMS [OR: 0.37; 95% CI, 0.14 to 0.9, *p* = 0.042] but was ineffective in reducing the incidence of severe AMS [OR: 0.46; 95% CI, 0.14 to 1.41, *p* = 0.17]. Inhaled budesonide was also effective in maintaining SpO2 (SMD: 0.47; 95% CI, 0.09 to 0.84, *p* = 0.014) at high altitude. However, it was not effective in maintaining or improving pulmonary function at high altitude. Systematic-review found no adverse effects of budesoide in the dose used for prophylaxis of AMS.

**Conclusions:**

Our systematic review showed that prophylactic inhaled budesonide is effective in preventing mild AMS during emergency ascent but not effective in preventing severe AMS. Though statistically significant, authors recommend caution in interpretation of data and questions for further well designed randomized studies to evaluate the role of budesonide in prophylaxis of AMS during an emergent ascent.

## Background

Acute Mountain Sickness (AMS) is condition that occurs in non-acclimated individuals rapidly ascending to high-altitude. Above altitude of 2500 m, the barometric pressure falls and the decreased barometric pressures translates to a decreased partial pressure of alveolar oxygen (PAO2) and arterial oxygen (PaO2). This results in a hypoxic challenge to any individual ascending to higher altitude [1]. The normal compensatory response to hypobaric hypoxia is termed acclimatization, which begins within minutes of ascent but requires several weeks to complete [2, 3]. With rapid ascent, time for acclimatization is insufficient predisposing an individual to an increased risk of AMS [4]. The prevalence of AMS is correlated with increasing altitude and the risk is significant at altitude above 2500 m. The prevalence of AMS at 2500 m is amongst 9 to 25% which increases to 75% at altitude above 4500 m [1].

The major determinants of AMS are the altitude attained, individual susceptibility, rate of ascent and degree of acclimatization [5]. AMS consists of nonspecific symptoms that occur within 6–12 h of arrival to altitude above 2500 m [6]. The symptoms are usually most pronounced after the first night spent at a new altitude and resolve spontaneously when appropriate measures are taken. High-altitude headache is a primary symptom of AMS and occurs with lassitude, malaise, nausea and dizziness [6, 7]. No reliable genetic or physiologic markers are available to predict an individual’s susceptibility to altitude illness which includes AMS, high altitude pulmonary edema (HAPE) or high altitude cerebral edema (HACE). A gradual staged ascent with sufficient acclimatization can prevent AMS. But emergent circumstances requiring exposure to rapid atmospheric pressure changes – such as for climbers, disaster or rescue team procedures, and military operations – establishes a need for effective prophylactic mediations. Currently, no gold-standard medication exists for emergency ascent.

Literature has shown acetazolamide, a carbonic anhydrase inhibitor, takes at least 1 day prior to ascent to its render beneficial effects, and oral dexamethasone, though effective in emergency situations, is associated with serious systemic side effects including gastrointestinal bleeding and hypothalamic-pituitary-adrenal (HPA) hormonal impairment [8–11]. More recently, budesonide (BUD), an inhaled glucocorticoid with fewer systemic side effects, has been considered a potential preventative measure in emergency ascent. Studies evaluating effectiveness of BUD as a prophylactic agent for AMS have yielded mixed results [12–16]. It has been hypothesized that signals arising from hypoxic lungs causes inflammation and oxidative damage and increase capillary permeability in lungs and brain, which is thought to be responsible for development of AMS. BUD probably suppresses this signal from hypoxic lungs to brain and prevent oxidative damage. Inhaled BUD has also been shown to blunt the response of aldosterone to renin elevation by suppression of Angiotensin Converting Enzyme, by preserving the integrity of pulmonary endothelial membrane [17].

This systemic review and meta-analysis aim to analyze the incidence of AMS during emergent ascent of non-acclimatized individuals receiving inhaled BUD compared to those receiving placebo. As no current meta-analysis exists, our results contribute robust evidence to medical literature regarding inhaled BUD’s role as a prophylactic medication in emergency settings.

## Methods

Our systematic review and meta-analysis of inhaled BUD as a prophylactic measurement for Acute Mountain Sickness used the PRISMA (Preferred Reporting Items for Systemic review and Meta-Analysis) statement in conjugation with the PRISMA checklist and flow diagram, for manuscript format development [18].

### Literature search

The following databases served as sources for published studies prior to September 2018: PubMed, Google Scholar, Embase. Searches were conducted using the keywords “budesonide” or “inhaled budesonide” in combination with “high altitude”, “acute mountain sickness”, “emergent ascent”, or “rapid ascent”. Titles, abstracts, and full text were screened for study and report characteristics that matched eligibility criteria. Two independent reviewers screened and retrieved reports, with an additional investigator participating in review of study eligibility and inclusion in the meta-analysis. All potentially relevant reports were read independently by each author.

### Eligibility criteria

Eligible studies were selected based on the criteria mentioned below:

#### Study design

All studies were peer-reviewed, double-blinded, randomized control trials (RCT) comparing a placebo control group to inhaled BUD treatment group in the setting of altitude ascent.

#### Definition of Acute Mountain sickness

All studies included were required to use the Lake Louise Symptom Score Questionnaire (LLS) to evaluate AMS. This is a subjective tool used to analyze the severity of symptoms reported during ascent to high altitude. Symptoms include high altitude headache, loss of appetite or nausea, dizziness, fatigue or lassitude and insomnia, each component score ranging from 0 to 3. Score of ≥3 was classified as mild AMS, 3–5 as moderate AMS and score ≥ 5 as severe AMS [19]. The new Lake Louise score was introduced in 2018, which does not include sleep as its component and is not considered for the purpose of this metaanalysis.

#### Participants

All studies were required to include participants that were not acclimatized prior to participation.

#### Dosage of budesonide

All studies were required to report definitive dosing intervals and concentrations of inhaled BUD.

#### Objective outcomes

Included studies were to objectively assess the efficacy of BUD in rapid ascent through outcomes such as: incidence of AMS, pulmonary function tests, blood hemoglobin-oxygen saturation on pulse oximetry (SpO2), and adverse event rates.

### Data abstraction

Data was manually extracted by investigators from eligible studies. The following variables were included: first author, type of design, site of study, year of publication, trial registration number, sample size, mean age and body mass index (BMI) of participants, incidence of AMS, pulse oximetry blood-oxygen saturation (SpO2), pulmonary function (variable measures), starting altitude, maximum altitude, mode of ascent, ascent duration, drug dosages, inclusion and exclusion criteria used by each study.

### Outcome measures

Our primary objective was to assess the efficacy of inhaled BUD on preventing AMS. Our primary outcome measure was incidence of mild and severe AMS [19]. The secondary outcomes were pulmonary function, SpO2 at maximum altitude and incidence of adverse effects.

### Assessment of methodological quality

Studies were reviewed independently by two investigators using the guidelines provided by the Cochrane Collaboration tool for assessing risk of bias. All studies were assessed for random sequence generation, allocation concealment, blinding of participants, incomplete data outcome, and selective outcome reporting [20].

### Statistical analysis

Statistical analysis was performed using Comprehensive Meta-Analysis software (CMA 3.3, Biostat, Englewood, NJ, 2014). Heterogeneity was estimated using Cochrane Q and I^2^ statistics. When significant heterogeneity was present, we selected the random-effects model to calculate the effects size, else fixed-effects model was used. Meta-analysis for the dichotomous data (AMS incidence) was performed using a random effects model with a treatment effect expressed as OR with 95% confidence intervals. The extracted data from the continuous data set was employed to obtain the standard mean difference (SMD) and 95% confidence intervals using the random effects model. The offset between the studies was estimated using Begg’s test and Egger’s test.

## Results

### Literature search and data extraction

**Fig. 1:**
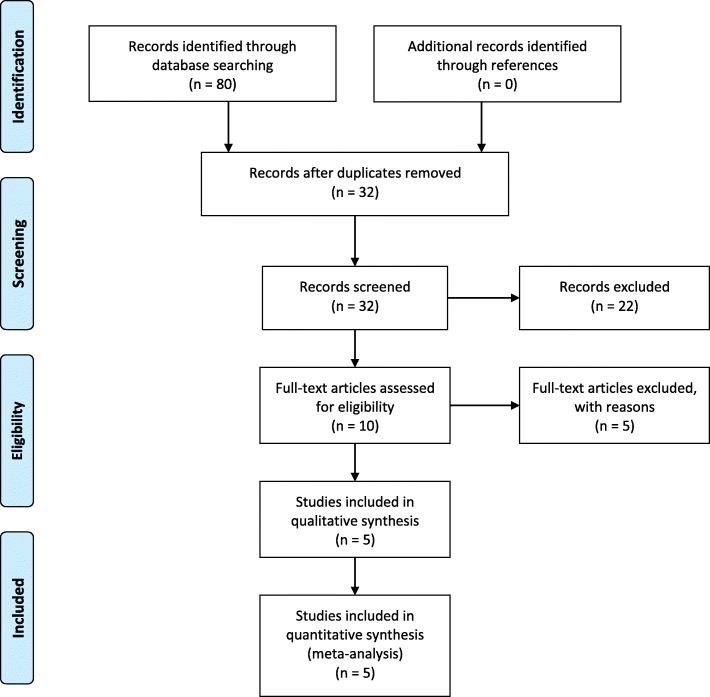
A PRISMA Flow Diagram representing the flow of literature search and selection done in the course of this Systematic Review

In total, 80 articles were identified after thorough database search. After exclusion of duplicates and those not meeting inclusion criteria, five studies were reviewed for data collection. Figure 1 shows the results of our literature search and selection. The characteristics of each included study discussed below are summarized in Tables 1 and 2.

**Table 1:** Key methodological characteristics of included studies

Study	Year	Study site	Trial registration number	Treatment groups and Sample size	Maximum Altitude	Starting altitude	Ascent mode	Total ascent duration	AMS definition	Severe AMS definition
**Zheng et al.**	2014	Litang County, Sichuan Province, China	ChiCTR-PRC-13003296	BUD (*n* = 42). DEX (*n* = 39). Placebo (*n* = 43).	4200 m	650 m	Car	4 days	LLS ≥ 3 with headache	LLS ≥ 5
**Chen et al.**	2015	Lhasa, Tibet, China	ChiCTRPRC-12,002,748	BUD (n = 20). PRO (n = 20). B/F (*n* = 20). Placebo (n = 20).	3700 m	500 m	By air	2.5 h	LLS ≥ 3 with headache	LLS ≥ 5
**Berger et al.**	2017	Capanna Regina Margherita, Monte Rosa, Italy	NCT02811016	BUD 200 μg (*n* = 16). BUD 800 μg (n = 17). Placebo (*n* = 17).	4559 m	1130 m	Rope way and Hiking	20 h	LLS ≥ 5 plus AMS-C score ≥ 0.70. However, separate analysis using LLS ≥ 3 with headache was also done. We included latter for analysis.	We included LLS ≥ 5 plus AMS-C score ≥ 0.70 for analysis of severe AMS.
**Lipman et al.**	2017	White Mountains of California, USA	NCT02604173	Placebo (n = 35). AZ (*n* = 35). BUD (*n* = 33).	3810 m	1240 m	Car and Hiking	4 h	LLS ≥ 3 with headache	LLS ≥ 5
**Wang et al.**	2018	Litang County, Sichuan Province, China	CHiCTR-PRC-16008441	I/S (*n* = 30). BUD (*n* = 31). SAL (*n* = 30). Placebo (n = 30).	4000 m	2000 m	Car	3 days	LLS ≥ 3 with headache	LLS ≥ 5

Budesonide: *BUD*

Dexamethasone: *DEX*

Procaterol: *PRO*

Budesonide / formoterol: B / F

Acetazolamide: *ACZ*

Ipratropium bromide / salbutamol: *I / S*

Salbutamol sulfate: *SAL*

**Table 2:** Characteristics of patients and interventions in each included study

Study	Study population	Exclusion criteria	Doses	Drug administration and assessment of AMS	Age in years (mean ± SD)	BMI (mean ± SD)
**Zheng et al.**	Non-Tibetan healthy young male lowland residents (18–35 years old).	HA (> 2500 m) exposure history in the past year; severe organic diseases; contraindications of budesonide or dexamethasone.	Budesonide: 200 μg per inhalation, bid. Dexamethasone: 4 mg, bid. Placebo.	Drugs started 1 day before ascent and continued for 2 days after high altitude exposure. AMS assessed 4 days after high altitude exposure (2 days after last inhalation)	Budesonide: 20.39 ± 2.40. Dexamethasone : 20.78 ± 2.30. Placebo: 20.52 ± 2.35.	Budesonide: 21.32 ± 2.28. Dexamethasone: 21.13 ± 1.86. Placebo 20.95 ± 1.95.
**Chen et al.**	Lowland residents at or below 500 m, healthy, and 18 to 35 years of age.	HA (> 2500 m) exposure history in the past year or organic diseases or psychological or neurological disorders.	Budesonide: 100 μg per inhalation, two inhalations bid. Procaterol: 25 μg bid. Budesonide/formoterol: 160 μg budesonide/4.5 μg formoterol per inhalation, one inhalation, bid. Placebo.	Drugs started 3 days before ascent and stopped after high altitude exposure. AMS assessed 1 day after high altitude exposure (1 day after last inhalation).	Budesonide : 21.85 ± 3.23. Procaterol: 20.30 ± 2.03. Budesonide/formoterol: 20.60 ± 2.76. Placebo: 21.65 ± 3.31	Budesonide: 20.98 ± 2.21. Procaterol: 21.00 ± 1.44. Budesonide/formoterol: 21.64 ± 1.49. Placebo: 22.15 ± 2.94.
**Berger et al.**	Healthy, non-smoker, non-acclimatised lowlanders were included in the study	Spent time at altitudes > 2000 m within the past 4 weeks before the study, took any regular medication	Budesonide 200 μg: bid. Budesonide 800 μg: bid. Placebo.	Drugs started 1 day prior to ascent and continued for 4 days (2 days after high altitude exposure). AMS assessed on last day of inhalation (2 days after high altitude exposure).	Budesonide 200 μg: 38 ± 11. Budesonide 800 μg: 38 ± 11. Placebo: 36 ± 12.	Budesonide 200 μg: 24.0 ± 2.1. Budesonide 800 μg: 22.5 ± 2.2. Placebo: 22.8 ± 2.5.
**Lipman et al.**	Healthy, low landers < 1240 m (4,100 ft), able to complete a moderately strenuous hike at high altitude	Younger than 18 years old or over 65, pregnant or considered pregnant, living or sleeping at an altitude of more than 1240 m (4,100 ft) last week, taking diuretics, steroids, acetazolamide or NSAIDs a week before the study, allergy to study drugs or a dangerous condition, which did not allow to travel at high altitude	Placebo. Acetazolamide: 125 mg PO bid. Budesonide: 180 μg per inhalation bid.	Drugs started on morning of ascent day and AMS assessed on evening after high altitude exposure.	Placebo: 32 ± 7. Acetazolamide: 33 ± 9. Budesonide: 33 ± 10.	Placebo: 24 ± 2.6. Acetazolamide: 24.1 ± 1.93. Budesonide: 22.7 ± 2.1.
**Wang et al.**	Healthy young male who lived a long term in 2000 m (18–28 years old).	HA (> 2500 m) exposure history in the past year; severe organic diseases or psychological or neurological disorders; contraindications of study drugs; other unsuitable conditions	Ipratropium bromide/salbutamol: 0.5 mg ipratropium bromide/3 mg salbutamol sulfate per inhalation, bid. Budesonide: 2.0 mg per inhalation, bid. Salbutamol sulfate: 5.0 mg per inhalation, bid. Placebo.	Drugs started on day of ascent and continued till high altitude exposure (3 days). AMS assessed after 3 days of high altitude exposure.	Ipratropium bromide/salbutamol: 21.89 ± 2.78. Budesonide: 21.35 ± 3.05. Salbutamol sulfate: 21.25 ± 2.35. Placebo: 22.83 ± 2.74.	Ipratropium bromide/salbutamol: 21.55 ± 1.31. Budesonide: 21.94 ± 2.11. Salbutamol sulfate: 21.40 ± 1.68. Placebo: 21.73 ± 2.25.

### Study design

**Fig. 2:**
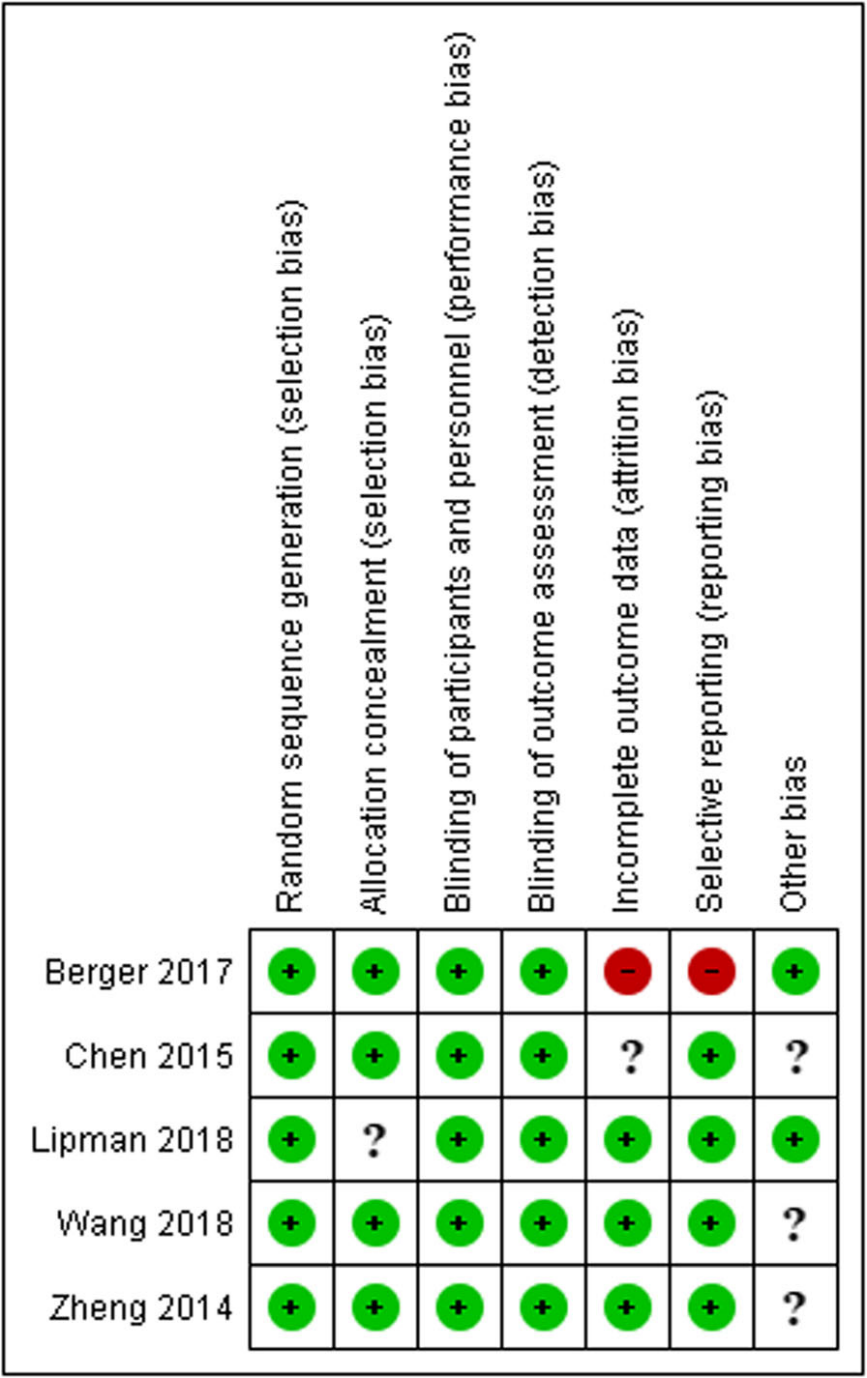
Risk of bias graph. “-” indicate high risk of bias, “+” indicate low risk of bias and “?” indicate unclear risk of bias

All included studies were randomized, double-blind, placebo-controlled trials comparing inhaled BUD with other control groups including placebo for the prevention of AMS. However, there was considerable heterogeneity in terms of study design. Three trials were registered in China and sponsored by Chinese institutions and two were registered in the USA including one sponsored by Italian institution. Three studies were conducted at an altitude of more than 4000 m. The starting height varied from 400 m to 2000 m. The method of ascent was through a car, air, cable car, and hiking. Some of the studies used a combination of the aforementioned methods. The ascent duration ranged from 2.5 h to 5 days. The methodological characteristics of all the included studies based on the guidelines provided by the Cochrane Collaboration tool for assessing risk of bias are summarized in Fig. 2. “-” indicate high risk of bias, “+” indicate low risk of bias and “?” indicate unclear risk of bias.

### Treatment groups

In all included RCTs, inhaled BUD was compared with several control groups, including the placebo group. In a study by Zheng et al., there were three treatment groups, viz. BUD, Dexamethasone (DEX) and placebo with 42, 39, and 43 subjects, respectively [12]. In a study by Chen et al., there were four treatment groups, BUD, Procaterol (PRO), budesonide / formoterol (B / F) and placebo with 20 subjects in each group [13]. In a study by Lipman et al. three treatment groups were included BUD, acetazolamide (ACZ) and placebo with 33, 35 and 35 subjects, respectively [15]. Berger et al. had two groups of BUD, 200 μg and 800 μg, and the placebo group [14]. These groups included 16, 17, 17 subjects, respectively. A study by Wang et al. there were four groups, viz. Ipratropium bromide / salbutamol (I / S), BUD, salbutamol sulfate (SAL) and placebo with 31 subjects in BUD group and 30 in rest [16].

### Drugs and doses

For comparison with the control groups, including the placebo, two different doses of BUD (200 μg bid. and 180 μg bid.) were used. Berger et al. included two different doses of BUD i.e. 200 μg and 800 μg [14]. However, for uniformity and analysis we only included data of 200 μg group. Doses of the control groups are shown in Table 2.

### Recruitment

Zheng et al. recruited non-Tibetan healthy young men of age 18–35 years, residing in lowland [12]. Chen et al. recruited healthy, lowland residents of age 18 to 35 years, residing at or below 500 m altitude [13]. Lipman et al. recruited healthy, low landers residing below 1240 m (4,100 ft.) who were able to complete moderately strenuous hike at high altitude [15]. Berger et al. recruited healthy, non-smoker, non-acclimatized lowlanders [14]. Wang et al. recruited healthy young male aged 18–28 years who lived a long term in 2000 m [16]. Exclusion criteria used during recruitment of the study subjects are tabulated in Table 2.

### End-point assessment

**Mild AMS:** All studies used LLS ≥ 3 with headache as mild AMS criteria. Meta-analysis for incidence of mild AMS was done using LLS ≥ 3 with headache criteria.

**Severe AMS:** Berger et al. did not classify severe AMS in their study. However, all other studies used LLS ≥ 5 as criteria for severe AMS. However Berger et al. used LLS ≥ 5 with an AMS-C score ≥ 0.7 for diagnosis of AMS in general. This criteria was considered equivalent to LLS ≥ 5 criteria and meta-analysis was done for severe AMS.

Pulmonary function was evaluated by different methods. In a study by Zheng et al., forced vital capacity (FVC), FVC %Pred. (percentage of the predicted value), forced expiratory volume in one second (FEV1), and FEV1%Pred. were achieved with a portable spirometer. In a study by Chen et al., vital capacity (VC) and FEV1 were measured using a portable spirometer. Berger et al. measured FEV1 and FVC using a portable spirometer. Lipman et al. and Wang et al. did not measure pulmonary function at altitude.

### Outcomes

#### Prevention of mild AMS

**Fig. 3:**
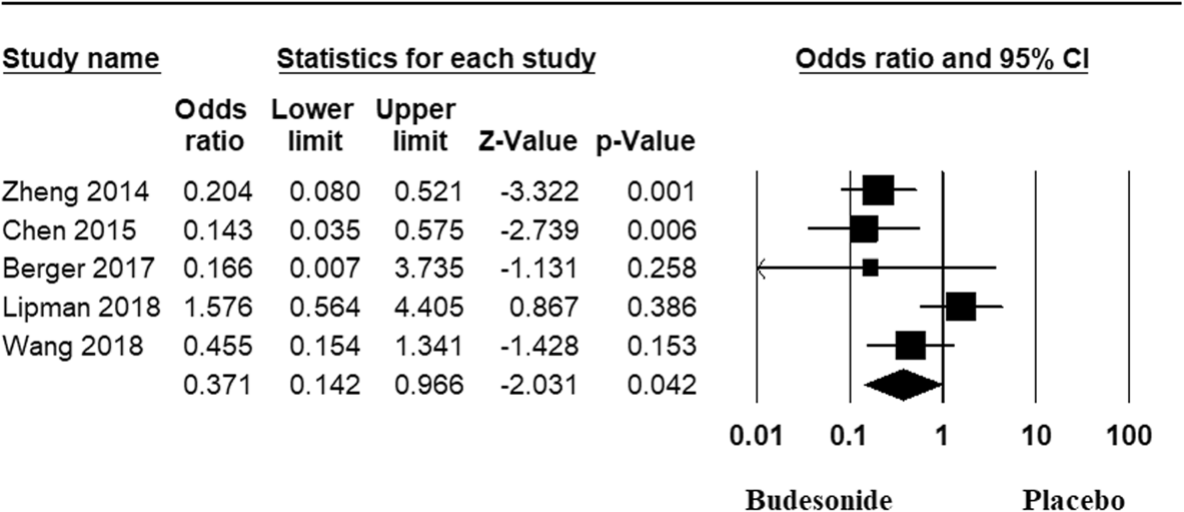
Forest plot with 95% CI for incidence of AMS. The area of each square is proportional to the study’s weight in the meta-analysis, while the diamond shows the pooled result. The horizontal lines through the square illustrate the length of the confidence interval. The width of the diamond serves the same purpose. The overall meta-analysed measure of effect is imaginary vertical line passing through diamond. If result estimates are located to the left, it means that the outcome of interest (incidence) occurred less frequently in the intervention group than in the control group

After performing meta-analysis, we found that at high altitude, inhaled BUD was effective in reducing the incidence of mild AMS [OR: 0.37; 95% CI, 0.14 to 0.9, *p* = 0.042]. There was significant heterogeneity between studies (I^2^ = 64.49%). The forest plot of the result in random effects model is demonstrated in Fig. 3. There was no evidence of publication bias by Begg’s test (*p* = 1) and Egger’s test (*p* = 0.67). We performed subgroup analysis to estimate the effect of various subgroups on incidence of mild AMS. Inhaled BUD was effective in reducing the incidence of mild AMS in subgroup of studies done in China [OR: 0.24; 95% CI, 0.13 to 0.46, *p* = 0.000, I^2^ = 0%] but not in studies from Europe and USA [OR: 0.84; 95% CI, 0.11 to 6.08, *p* = 0.86, I^2^ = 44.7%].

#### Prevention of severe AMS

**Fig. 4:**
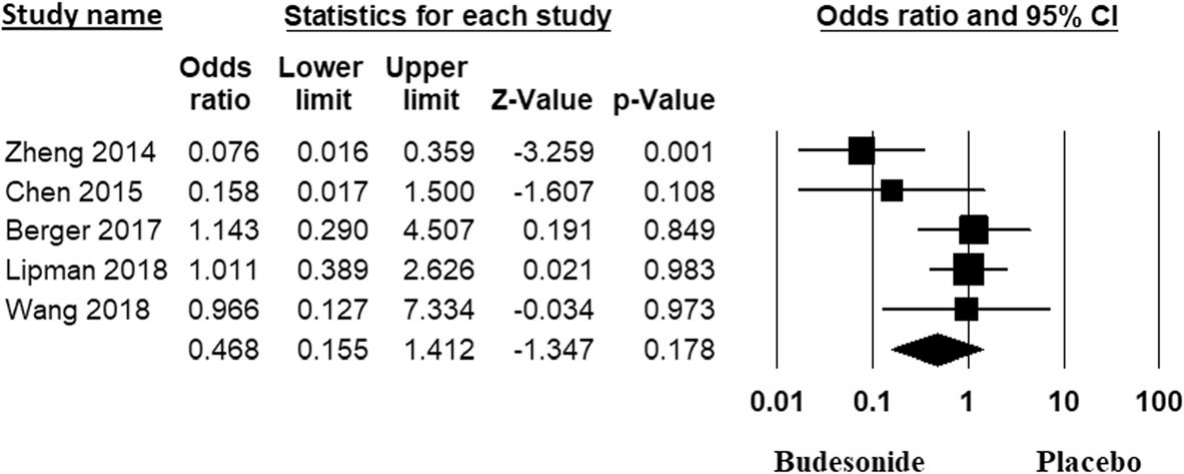
Forest plot with 95% CI for incidence of severe AMS

We found that, inhaled BUD was not effective in reducing the incidence of severe AMS [OR: 0.46; 95% CI, 0.14 to 1.41, *p* = 0.17]. There was significant heterogeneity between studies (I^2^ = 61.39%). The Forest plot of the result in random effects model is demonstrated in Fig. 4. There was no evidence of publication bias by Begg’s test (*p* = 0.22), and Egger’s test (*p* = 0.4). On subgroup analysis, we found that Inhaled BUD was effective in reducing the incidence of severe AMS in subgroup of studies done in China [OR: 0.2; 95% CI, 0.04 to 0.96, *p* = 0.04, I^2^ = 47.7%] but not in studies from Europe and USA [OR: 1.05; 95% CI, 0.48 to 2.3, *p* = 0.89, I^2^ = 0%].

#### SpO2 and pulmonary function

**Fig. 5:**
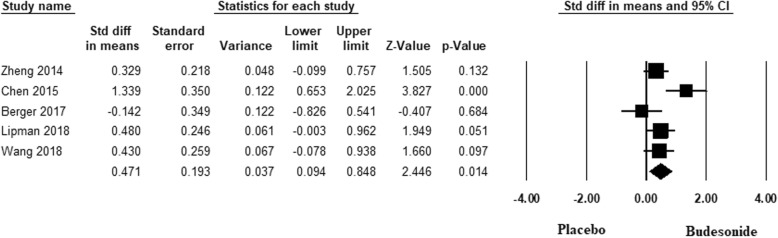
Forest plot depicting the standardized mean difference (SMD) and its 95% confidence interval for SpO2. The square shows the SMD for each study. The diamond at the bottom of the graph shows the average effect size of included studies

We found that inhaled BUD was effective in maintaining SpO2 (SMD: 0.47; 95% CI, 0.09 to 0.84, *p* = 0.014, I^2^ = 58.62%) at high altitude. Forest plot of the result in random-effects model is demonstrated in Fig. 5. There was no evidence of publication bias by Begg’s test (*p* = 0.85) and Egger’s test (*p* = 0.68). We found that inhaled BUD was effective in maintaining SpO2 (SMD: 0.643; 95% CI, 0.09 to 1.18, *p* = 0.02, I^2^ = 68.39%) at high altitude in subgroup of studies done in China. However, it was ineffective in maintaining SpO2 (SMD: 0.218; 95% CI, − 0.38 to 0.82, *p* = 0.47, I^2^ = 52.87%) at high altitude in subgroup of studies done in Europe and USA.

Since all included studies used different methods to assess pulmonary function and some studies did not assess pulmonary function, we were unable to conduct a meta-analysis on this. In the study by Zheng et al., the spirometric parameters were similar among various study groups at sea level. In every group, FVC and FVC % Pred. dropped after high-altitude exposure, while FEV1 and FEV1% Pred. did not change significantly. The inhaled BUD group had significantly smaller degree of decrement (∆ FVC and ∆FVC % Pred.) than the placebo group (both *P* < 0.05). In the study by Cheng et al., there were no significant differences in VC or FEV1 among the treatment groups at 20 h after exposure to high altitude. Further analysis also showed no differences between subjects with and without AMS in each group. Berger et al. showed that, FVC, FEV1, and the ratio between FEV1 and FVC were not different among groups at high altitude at any point of time. Based on these findings, it can be said that inhaled BUD is no better than placebo in preserving pulmonary function at high altitude.

#### Adverse effects

Zheng et al., Lipman et al., Cheng et al. and Wang et al. reported no adverse reactions related to the study drug in subjects from the inhaled BUD group. Berger et al. reported that plasma levels of adrenocorticotropic hormone and cortisol, as well as urine excretion of cortisol within 24 h did not differ between the studied groups, indicating that inhaled BUD did not suppress the hypothalamic-pituitary-adrenal axis. Therefore, inhaled BUD might not have any systemic effects.

## Discussion

In this systematic review and meta-analysis, we critically appraised five randomized controlled trials for the effectiveness of inhaled BUD as a prophylactic agent for AMS during emergent ascent. At high altitude, BUD was found to be effective in reducing the incidence of mild AMS during emergent ascent. However, on subgroup analysis, inhaled BUD was effective in reducing the incidence of mild AMS in subgroup of studies done in China but not in studies from Europe and USA. A possible explanation is in the difference between method of ascent and total ascent duration. All three studies by Chinese colleagues used passive method of ascent by car [12, 16] or by air [13] and 2 out of 3 studies by Chinese colleagues had an ascent duration beyond 48 h [12, 16]. Another explanation could be the difference between mean ages of subjects included in the study. Studies from China had mean age of 20 [12] or 21 yr [13, 16]. However, the mean age in studies by Berger et al. and Lipman et al. were 38 and 33 years respectively [14, 15]. Increasing age is associated with reduced plasma concentrations of budesonide at 20 min [21]. The lung matures by age 20–25 years, and thereafter aging is associated with progressive decline in lung function and further the airways receptors undergo functional changes with age that make them less responsive to medication [22]. We found that inhaled BUD was not effective in reducing the incidence of severe AMS. However, on subgroup analysis, inhaled BUD was effective in reducing the incidence of severe AMS in subgroup of studies done in China but not in studies from Europe and USA. This result can be explained by the same assumption mentioned above.

BUD is one of the most frequently prescribed inhaled corticosteroids worldwide that has been used in the long-term management of chronic obstructive pulmonary disease and asthma. Our systematic-review found that BUD is not effective in maintaining or improving pulmonary function. A possible explanation for this finding is that the observed decrease in pulmonary function is caused by a reduced effort due to fatigue or symptoms of AMS that may impair the maximum effort that is critical to adequate pulmonary function testing. SpO2 was significantly higher in BUD group than in the placebo group at high altitude, which indicate that inhalation of BUD have a significant effect on pulmonary gas exchange. However, significance was lost when subgroup analysis was done including studies done outside China.

Though there were many similarities among studies, including dosage of the study drug, size of the cohorts and AMS assessment tools, high level of heterogeneity was ubiquitous in all of our analyses. Taking help from the subgroup analyses, we tried to explore the source of heterogeneity in ours study. We found that heterogeneity might have been introduced because of the methodological or demographic differences among studies.

Fall in partial pressure of oxygen at altitude causes a low ventilatory drive, impaired gas exchange due to interstitial pulmonary edema, and increased metabolism ultimately causing hypoxemia and tissue hypoxia [1, 2]. Hypoxemia results in an increase in cerebral blood flow, increase in vascular permeability through a higher oxidative stress or low-grade inflammation or increased expression of vascular endothelial growth factor (VEGF), venous outflow restriction, free radicals induced pump failure, lipid peroxidation, and destabilization of astrocyte membrane causing astrocyte swelling and hence causes cerebral edema [1, 23–25]. Furthermore, there is activation of nociceptors in trigeminovascular system due to pressure or distortion through cerebral edema and also due to release of nociceptive chemicals [1, 3]. Accordingly, drugs that mitigates cerebral edema, such as ACZ [26] or DEX [27, 28], are well tested and validated by various studies and are currently in use for prevention of AMS. However, ACZ is not considered effective for emergent ascent [11] and DEX have various systemic side effects [8–10]. Steroids like BUD seems to be a conceptually and clinically attractive option for AMS prevention. Accordingly, our meta-analysis of RCTs testing inhaled BUD for preventing AMS found that BUD is effective in preventing mild AMS during emergent ascent. But it was found to be ineffective in preventing severe AMS. However, authors recommend caution in interpretation of data as the weightage of studies done in China by Zheng and Wang et al. may have tilted the equation in favor of BUD contrary to studies elsewhere. The rate of ascent was very slow in Chinese studies, approximately 3–4 days allowing acclimatization and cannot be exactly considered as emergent ascent. Further, LLS score was taken after 4 days. LLS is highly subjective and authors believe that the result may have influenced by high incidence of AMS which may be attributed to fatigue and motion sickness following prolonged journey by car over a number of days. However, it suggests that BUD might be considered as prophylaxis of AMS if the ascent is slow and might be considered in patients who are allergic to ACZ.

This study has some limitations. First, some trials recruited a small number of patients in each treatment group. Second, most studies do not systematically describe adverse reactions, making it difficult to get a definitive conclusion about the incidence of these adverse events. Third, we were unable to compare different doses of BUD, so dose-related responses could not be found due to lack of data in the included studies. Fourth, the AMS diagnostic criteria among included studies were not uniform. Finally, LLS was recently revised in 2018 [29] and did not include insomnia as the standard for AMS. All studies included took place before 2018.

## Conclusion

Our systematic review showed that prophylactic inhaled BUD is effective in preventing mild AMS during emergency ascent but not effective in preventing severe AMS. Though statistically significant, authors recommend caution in interpretation of data as the weightage of studies done in China may have tilted the equation in favor of BUD contrary to studies elsewhere. The rate of ascent was slow and passive in Chinese studies and cannot be considered emergent ascent. This questions for further randomized studies to evaluate the role of BUD in prophylaxis of AMS in both emergent and slow ascents.

## Data Availability

Not applicable.

## Abbreviations

AMS: Acute mountain sickness
RCT: Randomized controlled trial
BMI: Body mass index
PRISMA: Preferred Reporting Items for Systematic Reviews and Meta-Analyses
BUD: Budesonide
DEX: Dexamethasone
PRO: Procaterol
B / F: Budesonide / formoterol
ACZ: Acetazolamide
SAL: Salbutamol sulfate
OR: Odds Ratio
SpO2: Arterial hemoglobin oxygen saturation
SMD: Standard mean difference
LLS: Lake Louise Symptom Score Questionnaire
FVC: Forced vital capacity
FEV1: Forced expiratory volume in one second
VC: Vital Capacity
